# Stress Monitoring Using Machine Learning, IoT and Wearable Sensors

**DOI:** 10.3390/s23218875

**Published:** 2023-10-31

**Authors:** Abdullah A. Al-Atawi, Saleh Alyahyan, Mohammed Naif Alatawi, Tariq Sadad, Tareq Manzoor, Muhammad Farooq-i-Azam, Zeashan Hameed Khan

**Affiliations:** 1 Department of Computer Science, Applied College, University of Tabuk, Tabuk 47512, Saudi Arabia; 2 Applied College in Dwadmi, Shaqra University, Dawadmi 17464, Saudi Arabia; salyahyan@su.edu.sa; 3 Information Technology Department, Faculty of Computers and Information Technology, University of Tabuk, Tabuk 47512, Saudi Arabia; 4 Department of Computer Science, University of Engineering & Technology, Mardan 23200, Pakistan; 5 Energy Research Centre, COMSATS University Islamabad, Lahore Campus, Lahore 54000, Pakistan; tareqmanzoor@cuilahore.edu.pk; 6 Department of Electrical and Computer Engineering, COMSATS University Islamabad, Lahore Campus, Lahore 54000, Pakistan; fazam@cuilahore.edu.pk; 7 Interdisciplinary Research Center for Intelligent Manufacturing & Robotics (IRC-IMR), King Fahd University of Petroleum & Minerals (KFUPM), Dhahran 31261, Saudi Arabia

**Keywords:** healthcare, IoT, machine learning, sensor, stress

## Abstract

The Internet of Things (IoT) has emerged as a fundamental framework for interconnected device communication, representing a relatively new paradigm and the evolution of the Internet into its next phase. Its significance is pronounced in diverse fields, especially healthcare, where it finds applications in scenarios such as medical service tracking. By analyzing patterns in observed parameters, the anticipation of disease types becomes feasible. Stress monitoring with wearable sensors and the Internet of Things (IoT) is a potential application that can enhance wellness and preventative health management. Healthcare professionals have harnessed robust systems incorporating battery-based wearable technology and wireless communication channels to enable cost-effective healthcare monitoring for various medical conditions. Network-connected sensors, whether within living spaces or worn on the body, accumulate data crucial for evaluating patients’ health. The integration of machine learning and cutting-edge technology has sparked research interest in addressing stress levels. Psychological stress significantly impacts a person’s physiological parameters. Stress can have negative impacts over time, prompting sometimes costly therapies. Acute stress levels can even constitute a life-threatening risk, especially in people who have previously been diagnosed with borderline personality disorder or schizophrenia. To offer a proactive solution within the realm of smart healthcare, this article introduces a novel machine learning-based system termed “Stress-Track”. The device is intended to track a person’s stress levels by examining their body temperature, sweat, and motion rate during physical activity. The proposed model achieves an impressive accuracy rate of 99.5%, showcasing its potential impact on stress management and healthcare enhancement.

## 1. Introduction

Through the implementation of remote patient monitoring, the healthcare landscape has undergone a profound transformation due to the advent of the IoT [1]. The continuous tracking of physiological data made possible by the combination of wearable sensors with IoT technology allows for the early detection of health disorders and preventative measures before they escalate. Stress monitoring with wearable sensors and the Internet of Things (IoT) powered by lithium-ion batteries (LiB) is a potential application that can enhance wellness and preventative health management. A promising application that can improve comfort and preventative health management is stress monitoring with LiB-based IoT and wearable sensors. Stress, a pervasive health concern with potential physical and psychological ramifications, can be effectively addressed through this approach. Stress monitoring, when performed thoroughly and responsibly, is the modern approach in the healthcare industry that may recommend significant evidence to both patients and healthcare providers. It enables people to actively manage their stress and gives healthcare professionals the ability to provide individualized interventions and support. The scope of the IoT extends far beyond healthcare, encompassing domains such as energy efficiency, transportation, agriculture, logistics, and more [1]. However, its impact on healthcare, bolstered by the synergy of IoT and Artificial Intelligence (AI), has been particularly transformative [2]. This convergence has facilitated the transition of medical testing and healthcare services from hospitals to homes, thereby democratizing access to medical equipment for both individuals and healthcare professionals [2]. The amalgamation of mobile sensors with IoT infrastructure enhances data accuracy, and the fusion of an Android app with IoT technology enhances the usability of medical devices. The ripple effect of IoT is poised to be especially profound in the medical sector, promising an elevation in the overall quality of life [2]. In both daily life and healthcare, the applications of IoT and AI are manifold. As the utilization of the Internet has surged exponentially, conventional patient service methodologies have given way to electronic healthcare systems, leading to a decline in traditional modes of communication [3]. In recent times, IoT technology has empowered patients and healthcare practitioners to access cutting-edge medical equipment and resources. The benefits of AI and IoT extend across various realms, including mechanical automation, remote monitoring, convenience, financial efficiency, and enhanced patient satisfaction within healthcare applications [3]. For a sensor to qualify as a constituent of the IoT healthcare system, it must fulfill three core criteria. Firstly, it should be capable of monitoring pulse-related processes such as blood glucose levels, ECG, and oxygen levels. Secondly, it must possess the capacity to detect and gather environmental data like temperature, light, and precipitation. Thirdly, it should be equipped to autonomously transmit data, either dynamically or via an alternative mechanism, to a centralized controller. Following the completion of its designated task, the sensor should transition into an interactive mode, promptly alerting medical professionals for swift action [4]. The versatility of DNA origami extends not only to construction, transportation, and computation on two- and three-dimensional surfaces but also as a significant component of nanotechnology [5,6,7].

Progressive analyses of electronic health records (EHRs) and medical imaging have empowered researchers to enhance healthcare systems through innovative means. While healthcare apps and services are inherently geared toward meeting user needs, the extent of their development hinges on the capabilities and expertise of their developers. Recent explorations have delved into a diverse array of applications employing convolutional neural networks and other machine-learning methodologies. Notably, these techniques have been employed for accurate grading of alcohol dependence, estimation of accident severity, and recognition of emotions through technology [8,9]. The integration of the IoT and artificial intelligence has ushered in significant enhancements in daily life and healthcare. The conjunction and convergence of wearable sensors, IoT, and machine learning (ML) enable healthcare practitioners to diagnose and intervene in patients’ conditions at an early stage, thereby optimizing health outcomes. The multifaceted benefits of IoT devices, encompassing electronic information management, controlled communication, and system processing, have made them a focal point in medical applications due to their convenience, cost-efficiency, and augmented patient satisfaction.

Stress, a pervasive health concern, can be identified and managed effectively through the continuous monitoring of physiological signals via wearable sensors [10]. Recent advancements in IoT and machine learning have been instrumental in enhancing stress monitoring. Notably, wearable sensor systems have been developed to detect and track stress by analyzing physiological parameters like skin conductance and heart rate variability alongside contextual factors such as location and activity levels [11]. The multidisciplinary field of machine learning (ML) heavily relies on visualization, optimization, and theories of probability and decision-making. In contrast to studying each trait or feature separately, machine learning algorithms can handle enormous volumes of data efficiently and allow researchers to find patterns by simultaneously examining a mixture of qualities from the datasets. One of the key characteristics responsible for the enormous success of ML tools is their capacity to recognize a hierarchy of features and infer generalized trends from given data.

The integration of ML algorithms has facilitated the analysis of wearable sensor data, culminating in personalized feedback and interventions for users. The employment of ML and IoT for stress monitoring holds the possibility to enhance efficacy and accuracy. ML methods enable real-time analysis of acquired data, allowing for the early diagnosis and proceeding of stress-related conditions that could otherwise adversely impact overall health and quality of life. The transformative potential of IoT and ML in the healthcare sector is immense, encompassing continuous physiological signal monitoring, personalized interventions, and feedback mechanisms [12].

This paper aims to integrate the assessment of human stress levels through physical activity by employing ML technologies, wearable sensors, and IoT. This study identifies stress levels by monitoring indicators like human body humidity, temperature, and step count. With this integrated strategy, individuals may monitor their stress levels in real time and receive individualized feedback and control over how they react to stress.

## 2. The Literature Review

A set of essential characteristics for an ideal sensor includes qualities such as precision, sensitivity, linearity, repetition, reproducibility, drift, calibrating, and fast response [13]. When ML techniques are used, a positive feedback loop may be created, allowing for ongoing improvements in the therapeutic interventions provided to specific patients [14]. Almost any digital device, from wearable to other hardware kinds, can serve as an IoT device and be useful in a wide range of societal areas [15]. A comprehensive review of stress detection research is provided in a scholarly article [16]. Additionally, a study has found that muscle-to-muscle communication is influenced during periods of stress [17]. The specific issue of stress experienced while driving is addressed in another publication [18]. In [19], a stress monitoring approach is proposed that leverages data collected from a smartwatch. Furthermore, ref. [20] introduces a non-invasive method for stress monitoring. Another research paper [21] suggests a stress monitoring system that utilizes cortisol as a biomarker. However, it is important to note that none of these sources discuss the concept of “smart sleep” or include mechanisms for stress control, secure data transfer, or storage. In the context of sleep patterns, ref. [22] presents a study involving participants and utilizes existing wearable devices such as [23,24]. Furthermore, a few non-wearable solutions for sleep regulation are also available [25]. However, it is worth mentioning that these studies do not adequately consider other physiological features and do not emphasize the significance of secure transmission and storage mechanisms for the collected data. According to Bone et al. [26], ML techniques and signal processing can be employed for mental health monitoring continuously. Their work also furnishes a concise assessment of the existing challenges associated with implementing these approaches. While clinical support is accessible, the scarcity of specialized professionals creates difficulties in overseeing all patient activities. Signal processing can be employed to simulate behavior, focusing on the most pertinent situations for making essential decisions.

The actions that trigger the signals picked up by the sensors are referred to as “stressors.” The edge, the fog, or the cloud are all viable locations for computing during the data preparation stage. Edge computing centers are located close to the sensor, and fog computing centers are located between the sensor and the cloud. Cloud computations are those performed using the Internet. To gauge stress levels across various scenarios, researchers have introduced several approaches. These methods involve integrating biofeedback procedures with gaming [27], utilizing mobile phones for tracking [28], and monitoring an individual’s linguistic expressions [29]. Biomarkers used to detect stress levels encompass ECG, skin conductance, respiration, and surface electrocardiography, as outlined in [30]. Other methodologies involve heart rate variability, as in [31], and functional Magnetic Resonance Imaging (fMRI) for stress detection, as detailed in [32]. Numerous use cases exist for a healthcare system in an IoT context, including but not limited to autonomous insulin infusion, sleep monitoring, and mental health monitoring. It is critical to discover solutions for stress detection because global awareness of their significance is at an all-time high.

This literature review suggests that there is still some disagreement over the most effective method of evaluating physiological stress. Despite employing the same physiological indicators and classifiers, the classification accuracy attained by various studies differed substantially. Additionally, the specificity and sensitivity of stress-related biophysiological measures like heart rate and respiration rate were not known in the research [33,34]. Moreover, decision trees, random forests, and XGBoost are frequently used for stress prediction as a machine learning classifier [35]. Here, the research gaps are summarized as follows:

- Absence of User-Friendly Stress Detection Wearables: The nonexistence of user-friendly wearable devices explicitly designed for stress detection obstructs user engagement and the acceptance of stress management solutions;
- Automated Stress Detection and Classification Missing: Many automated systems lack robust methodologies for the automatic identification and categorization of stress levels, thereby limiting their effectiveness in timely interventions;
- Limited Incorporation of Multiple Stress Detection Features: Several studies failed to consider the integration of various features for accurate stress detection and evaluation, potentially leading to incomplete or imprecise stress monitoring.

### Hypothesis for Stress Level Detection

Using the Stress-Track system, we put forth the following hypothesis:

*Hypothesis:* We believe that by observing changes in temperature and sweat during various physical activities, it is possible to monitor stress levels, ultimately identifying biomarkers indicative of chronic stress.

Mathematically, we can write an equation for this hypothesis as follows:

(1) $${Stress(t)=K}_{1}\times T(t)$$

where *Stress(t)* represents the stress level at time t. *T(t)* represents the change in temperature at time t. *K*_1_ is a constant that quantifies the relationship between temperature changes and stress levels.

*Background:* This theory is supported by the knowledge that engaging in physical activity causes the release of endorphins, also known as the “feel-good” hormone produced by the human brain [36]. Elevated stress levels can impede endorphin production, thereby diminishing the positive effects of physical activity. As the rate of steps taken per minute increases, so does the frequency of breaths per minute, heart rate, and overall stress on the body [37]. Moreover, heightened sweat levels in common areas like the palms and face correspond to an increase in an individual’s stress levels in a linear fashion [38]. Furthermore, the body’s temperature is influenced by blood circulation. Cold hands or feet often signify heightened stress levels, while warmer extremities indicate normal stress levels [39]. In the subsequent sections, we detail our proposed system design both at the architectural and sensor levels.

## 3. Proposed Method

**Figure 1:**
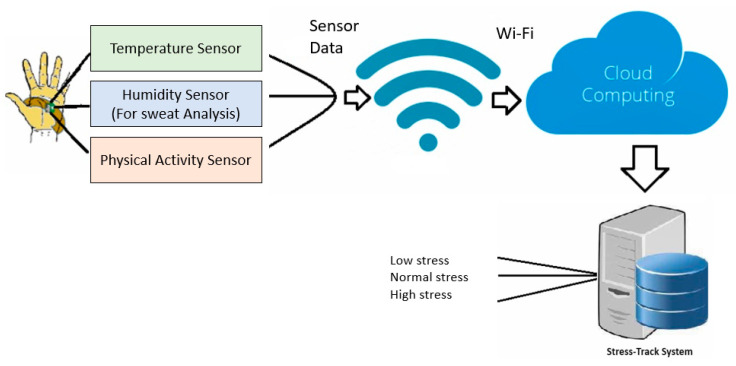
Proposed model.

We developed the stress detection system to cleverly monitor three essential metrics: body temperature; sweat reduction rate; and motion detection. This is in line with our research hypothesis. Figure 1 carefully depicts the *Stress-Track system’s* overall layout. By harnessing sensor data directly from the human body, our system engages in an intricate stress analysis through the utilization of machine learning located in the cloud. This advanced analytical process effectively categorizes stress levels into three distinct tiers: low; normal; and high.

Furthermore, our system integrates seamlessly with Wi-Fi technology, which, in turn, enables robust cloud connectivity. This dynamic connectivity feature serves a dual purpose: it facilitates the real-time storage of both current and historical stress levels, all within certain predetermined intervals. This architecture not only empowers the instant retrieval of stress data but also provides a comprehensive understanding of stress variations over time.

### 3.1. Stress-Track Sensing Wrist Band

Biosignals, which are time-dependent measurements of biological activities taking place within the human body, can be used to determine a person’s health. It has been demonstrated that biosignals are effective stress indicators [11,40].

#### 3.1.1. Body Temperature

Body temperature serves as a key indicator for a range of health conditions, both major and minor. By scrutinizing patterns in temperature fluctuations, it becomes possible to assess an individual’s physical and mental well-being. The temperature rate, denoting the pace at which body temperature changes over a specific timeframe, offers valuable insights. Contact temperature sensors, which collect temperature data when put on the body, and non-contact sensors, which measure infrared or optical radiation emitted from various body parts, are the two main categories into which temperature sensors fall [41]. Here, we used contact temperature sensors for accuracy and stability, which are designed to measure skin temperature. Skin temperature is commonly employed as a gauge for general thermal comfort or stress because it fluctuates more than core temperature does.

#### 3.1.2. Humidity Analysis

Sweat, a physical substance released through the skin’s pores in reaction to factors such as heat, physical activity, and emotional shifts, serves as an important physiological indicator. The body effectively becomes a variable resistor as the body’s sweat production increases due to a matching increase in the current flow between two electrodes [42]. To gauge sweat secretion levels, which are intricately governed by the human central nervous system, humidity-detecting sensors prove valuable. This monitoring of sweat volume has the potential to provide insights into stress and arousal levels experienced by the individual under observation. The variability in sweat gland activity holds significance across various biofeedback applications, encompassing areas like deception detection and emotion recognition [43]. While the usual sweating process is known as perspiration, excessive sweating falls under the classification of hyperhidrosis, often associated with emotional, professional, and societal stressors. In this specific study, the detection of sweat secretion on the palms is facilitated by the utilization of a humidity sensor.

#### 3.1.3. Step Count Analysis

Acceleration is the rate at which a given force causes a change in velocity. These forces that affect velocity changes can be static or dynamic. The number of steps taken by a person, as well as other actions like sitting, standing, walking, etc., are all counted using an accelerometer sensor.

### 3.2. Dataset

**Figure 2:**
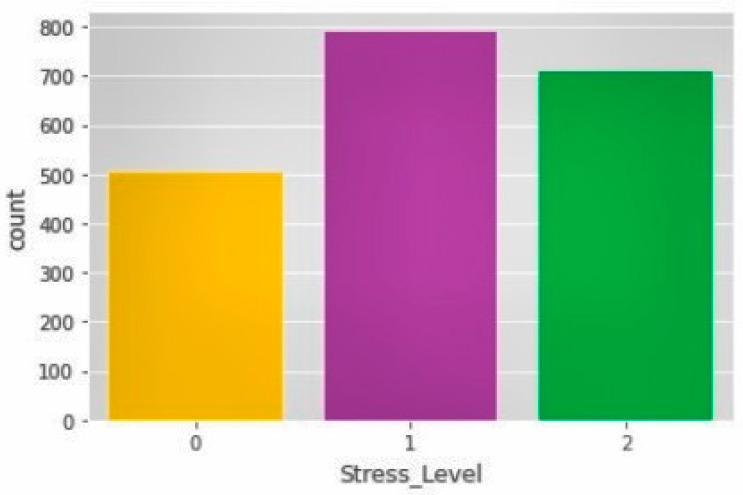
Stress level indication.

The dataset file “Stress-Lysis.csv” [11,44] contains information under the title “Humidity–Temperature–Step count–Stress levels”. This dataset focuses on identifying and evaluating stress levels in individuals based on their physical activity. It comprises a collection of 2001 samples, encompassing data related to human body temperature, body humidity, the number of steps taken by the user, and their corresponding stress levels. The stress levels are categorized into three groups: low; normal; and high stress. The dataset serves as a foundation for understanding the relationship between physical activity metrics and stress levels in different categories, as demonstrated in Table 1 and Figure 2.

In Table 1 above, the value 0 corresponds to “low stress”; 1 represents “normal stress”, and 2 signifies “high stress”.

### 3.3. Classifier

The machine learning model located in the cloud is utilized to detect the stress level; for this, we used the ensemble method of machine learning.

#### 3.3.1. Random Forest

The Random Forest (RF) is a tree structure formed by using a multitude of Decision Trees (DTs), where the branches are constructed based on randomly chosen parameters [45]. In mathematical terms, this can be represented as follows:

$$RF=\{{DT}_{1},{DT}_{2},\ldots \ldots {DT}_{N}\}$$

where $\left\{{DT}_{1},{DT}_{2},\ldots \ldots {DT}_{N}\right\}$ represent N individual Decision Trees in the Random Forest.

It works well as an ensemble learning strategy for bagging. In the context of ensemble learning, bagging can be expressed as follows:

Bagging (Bootstrap Aggregating): Given a training dataset D of size N, bagging creates B subsets $D_{1},D_{2},$……, $D_{B}$ of size N, each drawn with replacement from D.

Every DT in the RF algorithm makes a prediction about the class, and these predictions are pooled through voting to identify the class that receives the most votes. Mathematically, this can be represented as follows:

$$Class\_Prediction(RF)=Majority\_Vote\{{DT}_{1},{DT}_{2},\ldots \ldots {DT}_{N}\}$$

where Class Prediction (RF) is the final prediction made by the Random Forest, and Majority_Vote represents the process of selecting the class with the highest number of votes from the predictions of individual Decision Trees. This combination of predictions from various DTs frequently yields a prediction that is more accurate than any single prediction, which can be expressed using statistical concepts like ensemble variance reduction.

The number of trees in the used RF algorithm is fixed at 100, and subsets do not split if their size is smaller than five. This specifies the hyperparameters of the Random Forest:

Number of Trees (N) = 100 Minimum Subset Size for Splitting = 5

These hyperparameters control the size and behavior of the Random Forest model during training and prediction.

#### 3.3.2. Gradient Boosting (GB)

GB is indeed an efficient ensemble learning algorithm used for both classification and regression problems [46]. In mathematical terms, Gradient Boosting can be represented as follows:

$$GB=\{{DT}_{1},{DT}_{2},\ldots \ldots {DT}_{N}\}$$

where ${DT}_{1},{DT}_{2},\ldots \ldots {DT}_{N}$ represent N individual Decision Trees used in the Gradient Boosting ensemble.

The Gradient Boosting technique builds the ensemble of Decision Trees sequentially. It develops weak learners by trying to reduce the loss function. Mathematically, the update of the ensemble at each stage can be described as follows:

$${Ensemble}_{i}={Ensemble}_{\left\{i-1\right\}}+Learning\_Rate\times {DT}_{i}$$

where:

${Ensemble}_{\left\{i\right\}}$ represents the ensemble of trees at stage i.

Learning_Rate is a hyperparameter set to 0.1 in this case.

${DT}_{i}$ represents the $i^{th}$ Decision Tree added to the ensemble.

The number of estimators is set to 100 in the GB ensemble technique, which means that 100 Decision Trees are sequentially added to the ensemble during training. This hyperparameter controls the size of the ensemble and can affect the model’s complexity and performance.

#### 3.3.3. Stacked Ensemble Method (SEM)

Stacking is a powerful ensemble learning technique that combines the predictions of multiple machine learning algorithms to improve overall model performance. It involves the use of two levels: Level-0 and Level-1.

Level-0 (Base Models): In Level-0, individual machine learning models, often referred to as base models or predictors, are employed. These base models are trained on the same dataset, and each of them makes its own predictions. These predictions can be for classification or regression tasks.

Level-1 (Meta-Model): In Level-1, a meta-model is used to combine the predictions of various models acquired from Level-0. Instead of relying on simple techniques like hard voting or averaging, stacking takes a more sophisticated approach. It trains a meta-model using the Level-0 predictions as features to generate the final prediction or classification. The meta-model learns how to best weigh and combine the outputs of the base models. 

The process of stacking can be described as follows:

Level-0:

- Base Model 1 predicts: Prediction_1;
- Base Model 2 predicts: Prediction_2;
- Base Model 3 predicts: Prediction_3;
- ...

Level-1 (Meta-Model):

- Input features: [Prediction_1, Prediction_2, Prediction_3, …];
- Meta-model generates the final prediction: Final_Prediction.

**Figure 3:**
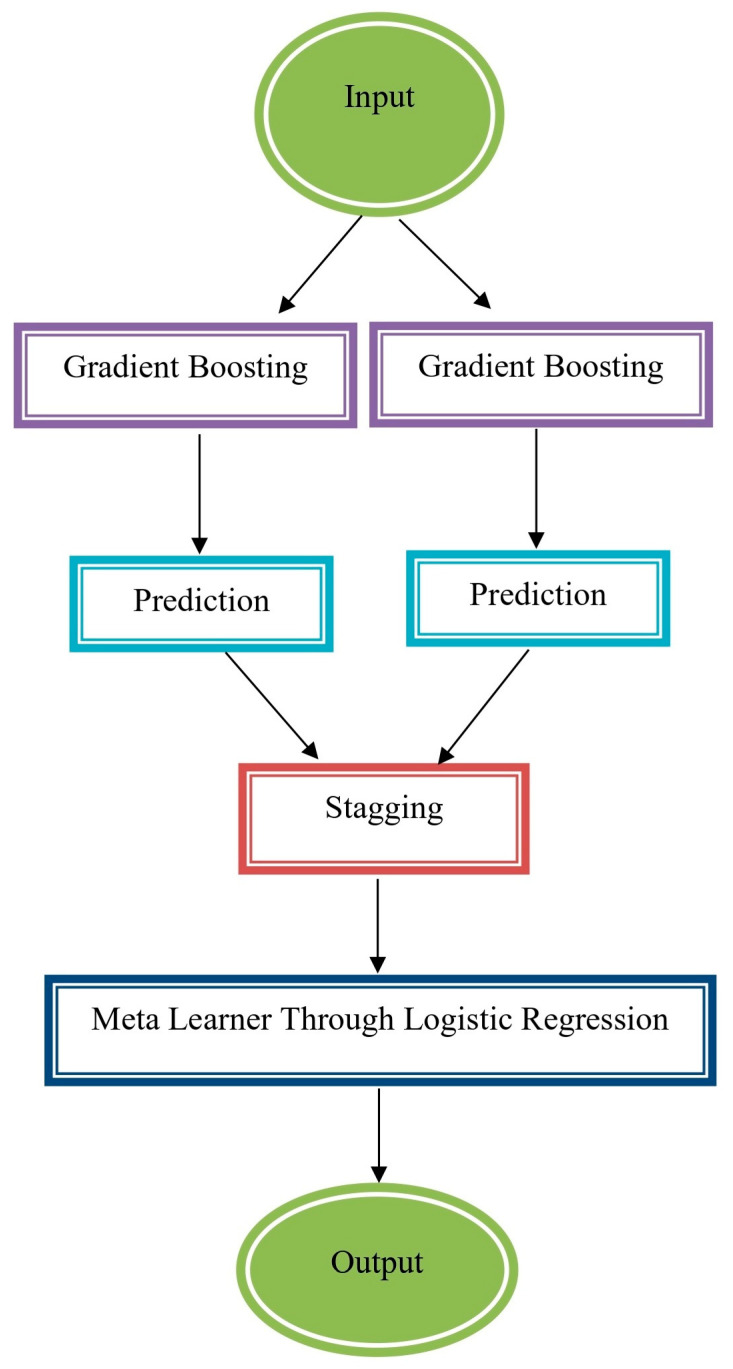
Stacking ensemble method.

The idea behind stacking is to leverage the diversity of the base models to capture different patterns and insights from the data. By training a meta-model on their predictions, stacking aims to improve the model’s performance, often achieving better results than individual base models or simpler ensemble methods. A generalized illustration of stacking ensemble method is provided in Figure 3.

The pseudo-code of the proposed Stress-Track sensor system and stacked ensemble method is described below:

A. Data Acquisition and Preprocessing
  i. Load the “Stress-Lysis.csv” dataset containing humidity, temperature, step count, and stress level;
  ii. Split the dataset into features (humidity, temperature, step count) and target variables (stress level);
  iii. Encode the categorical target variables (low, normal, high stress) into numerical values (0, 1, 2);
  iv. Perform any necessary data cleaning and preprocessing.
B. Stress-Track Sensing Wrist Band
  a. Body Temperature Measurement
    i. Select appropriate temperature sensors (contact or non-contact);
    ii. Collect temperature data from the body;
    iii. Analyze temperature patterns for health assessment.
  b. Humidity Analysis
    i. Utilize humidity-detecting sensors;
    ii. Monitor sweat secretion on the palms;
    iii. Analyze sweat levels for stress and arousal insights.
  c. Step Count Analysis
    i. Employ an accelerometer sensor;
    ii. Measure the individual’s step count.
C. Machine Learning Model
    i. Initialize the ensemble model (stacked ensemble);
    ii. Divide the dataset into training and testing sets.
  a. Base Models (Level-0)
    i. Train individual base models (e.g., Random Forest, Gradient Boosting) on the training data;
    ii. Generate predictions for stress levels on the testing data using base models.
  b. Meta-Model (Level-1)
    i. Collect predictions from the base models;
    ii. Train a meta-model (e.g., another Random Forest or Gradient Boosting) on the training data with base model predictions as features;
    iii. Use the meta-model to generate the final stress level predictions for the testing data.
D. Evaluation
  i. Assess the model’s performance using various metrics:
    - Accuracy;
    - Confusion matrix (true positives, true negatives, false positives, false negatives);
    - Precision;
    - Recall;
    - F1 measure.

## 4. Experimentation

### 4.1. Performance Matrices

Several measures, including accuracy, F1 measure, and confusion matrix, were used to assess the performance of the model used in this study.

- The percentage of accurate predictions made by the model is measured by accuracy;
- The confusion matrix is a table that compares the actual and anticipated classifications to summarize the presentation of a classifier.

The objective of this study was to categorize various levels of stress, and Table 2 reports the classification’s prediction measures. The assessments were made to see how effectively the models could categorize the stress levels.

The words true positive, false positive, true negative, and false negative are used to describe the parameters as they are being evaluated. Equations (2)–5 show how to obtain the formulas for accuracy, recall, and precision based on Table 2.

(2) $$Accuracy=\frac{\left(T^{-}+T^{+}\right)}{\left(T^{-}+F^{+})+(F^{-}+T^{+}\right)}$$

(3) $$Recall(R)=\frac{\left(T^{+}\right)}{\left(F^{-}+T^{+}\right)}$$

(4) $$Precision(P)=\frac{\left(T^{+}\right)}{\left(F^{+}+T^{+}\right)}$$

(5) $$Fscore=\frac{2\times (P\times R)\;}{\left(P+R\right)}$$

Moreover, the following parameters considered for this study are presented in Table 3.

The key attributes in this study are presented in Table 3. First, this study involved a total of 2001 sample size. Second, this study design was adopted as an “experimental design”. Third, this study established a level of significance set at 0.05.

### 4.2. Results and Discussion

IoT devices have been added to a few physical objects, allowing for real-time monitoring and data transfer across a variety of communication protocols, including Bluetooth and Wi-Fi. The heart rate, ECG, and EEG are a few instances of the crucial physiological data that these sensors are utilized to gather in the healthcare sector.

In this research, we conduct our experimental investigation using the “Stress-Lysis” dataset [11,44], and our evaluation revolves around the utilization of the stacking ensemble method. To comprehensively assess the effectiveness of the proposed approach, we employ a diverse range of metrics, including accuracy, the confusion matrix, precision, recall, and the F1 measure [47]. Accuracy serves as a holistic indicator of how effectively the classifier predicts stress levels in individuals during their sleep. This metric offers a global perspective on the model’s overall performance in capturing various stress levels accurately. The utility of the confusion matrix becomes evident as it empowers us to delve deeper into the classification outcomes. By analyzing the matrix, we can pinpoint the number of true positives, true negatives, false positives, and false negatives that the classifier generated. This level of granularity furnishes us with insights that go beyond the scope of accuracy alone. It unveils the model’s capability in correctly identifying positive and negative instances while revealing any potential areas where the classifier might be struggling. The synergy of these metrics ensures a robust and multifaceted evaluation of our approach, painting a more comprehensive picture of its performance in stress level prediction.

**Figure 4:**
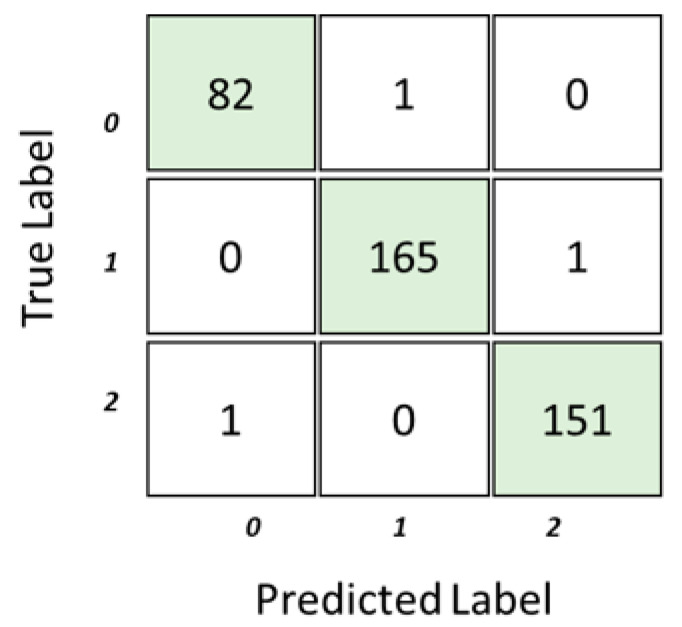
Confusion matrix.

The confusion matrix in Figure 4 offers a comprehensive overview of the model’s classification performance across different categories when applied to 20% of testing data. The number of examples that belong to each of these classes is shown in the matrix. The actual class is represented by each row, while the anticipated class is represented by each column.

In the first row (Class 0), there were 83 instances categorized as Class 0, and the classifier accurately identified 82 of them as Class 0. However, one instance was misclassified, where an instance from Class 0 was incorrectly labeled as Class 1. In the second row (Class 1), there were 166 instances that truly belonged to Class 1, and the classifier correctly identified 165 of them as Class 1. There was a single instance from Class 1 that the classifier mistakenly classified as Class 2. In the third row (Class 2), there were 152 instances that originally fell under Class 2. The classifier correctly identified 151 instances as Class 2. However, one instance from Class 2 was inaccurately classified as Class 0.

The presented metrics in Table 4, including accuracy, precision, recall, and F1 score, play a vital role in evaluating the performance of a classification model. Accuracy denotes the ratio of correctly predicted instances to the total, highlighting that the model achieved an impressive 99.5% accuracy in its predictions. Precision pertains to the accuracy of positive predictions; in this instance, it reveals that out of all instances predicted as positive, 99% were accurate, while 1% were false positives. Recall, also known as sensitivity, signifies the model’s ability to correctly identify positive instances, with 99% of true positives captured but 1% being missed. F1 Score harmonizes precision and recall, reflecting an overall balance; its value of 0.99 indicates a robust equilibrium between accurate positive predictions and the model’s capability to capture actual positives.

**Figure 5:**
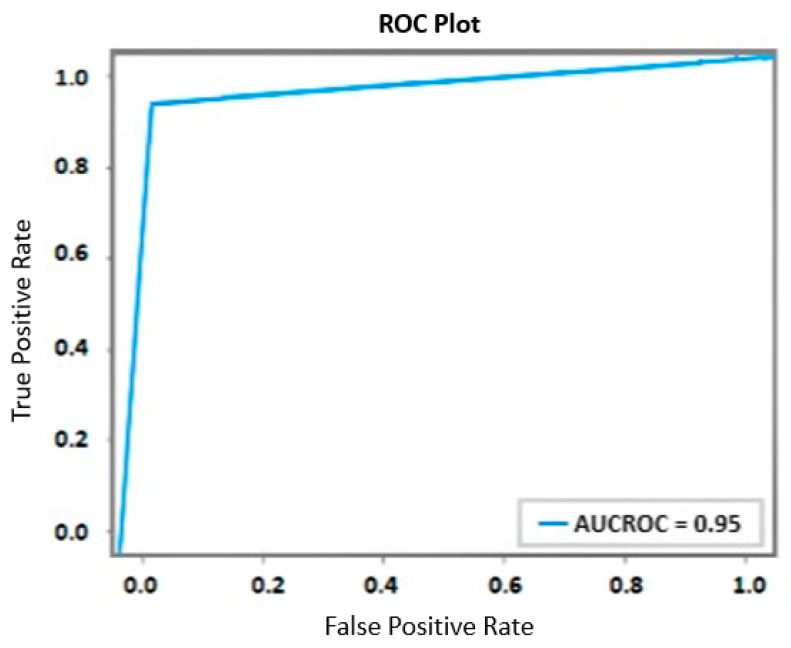
ROC Curve.

An ROC (Receiver Operating Characteristic) curve has been created to graphically show the performance outcomes. The trade-off between the TP rate and the FP rate is depicted graphically, enabling a thorough evaluation of the model’s predictive skills. Figure 5, where the x-axis indicates the false positive rate and the y-axis the true positive rate, displays this curve. This model is effective at differentiating between the classes, as evidenced by the derived Area Under the Curve (AUC) value, which is 0.95. This numerical value highlights the model’s ability to categorize occurrences into relevant groups in an efficient manner. The model’s accuracy in this task is supported by an AUC of 0.95, highlighting its ability to distinguish between positive and negative instances with accuracy.

## 5. Conclusions and Future Work

At present, the primary focus of individuals in our society is on financial survival, often leading them to disregard their well-being. The advent of intelligent sensors with the help of ML has the capability to consistently collect data, monitor an individual’s conduct, and predict the occurrence of a heart attack even before the patient feels its impact. Thus, a Stress-Track system is really important for keeping an eye on chronic stress from the beginning. This new system helps find out how much stress someone has. But it is not only about checking stress. The system keeps data safe by sending information about the body and stress over Wi-Fi to a secure place in the cloud. This makes it easier to collect and keep track of data.

The proposed system could help improve a person’s mental health and overall well-being. It can find stress and keep track of it accurately and dependably. This system is helpful and can make society healthier by dealing with stress in a good way. The achieved accuracy is 99.5%, which shows that it could really help with managing stress and making healthcare better. Compliance with health rules and standards ensures accurate management of sensitive health information. Overall, incorporating stress monitoring into battery-based wearable technology has the potential to revolutionize healthcare by delivering helpful data and promoting a proactive, individualized approach to healthcare services. Stress levels of patients may be remotely monitored, which enables medical professionals to assess the efficacy of treatment and modify plans as needed. Those who suffer from chronic ailments would particularly benefit from this.

Implementing our system in real-world scenarios may present challenges in terms of scalability and resource requirements. Ensuring a seamless integration of our sensor system into various environments and devices is a significant hurdle. Moreover, the effective deployment of this system in healthcare or other sectors would require careful consideration of regulatory and privacy issues. In future work and discussions, we will explicitly highlight the ethical considerations surrounding data usage in our system and the steps taken to protect user privacy. 

## Figures and Tables

**Table 1:** Demonstration of the employed dataset.

Humidity	Temperature	Step Count	Stress Level
21.33	90.33	123	1
21.41	90.41	93	1
27.12	96.12	196	2
27.64	96.64	177	2
10.87	79.87	87	0
11.31	80.31	40	0
18.16	87.16	88	1
28.2	97.2	162	2
14.25	83.25	61	0
26.13	95.13	168	2
23.61	92.61	200	2
19.37	88.37	117	1

**Table 2:** Prediction measures.

Measures	Definition
$T^{-}$	Accurate recognition of negative data
$T^{+}$	Accurate recognition of positive data
$F^{-}$	Incorrectly classifying negative data
$F^{+}$	Incorrectly classifying positive data

**Table 3:** Model’s parameters.

Sample Size	Study Design	Level of Significance
2001	Experimental design	0.05

**Table 4:** Classification model’s results.

Accuracy	Precision	Recall	F1 Score
99.5	0.99	0.99	0.99

## Data Availability

The dataset used in this research is available online.
